# Evaluation of the *Schistosoma mansoni* Y-box-binding protein (SMYB1) potential as a vaccine candidate against schistosomiasis

**DOI:** 10.3389/fgene.2014.00174

**Published:** 2014-06-11

**Authors:** Sílvia R. C. Dias, Mariana Boroni, Elizângela A. Rocha, Thomaz L. Dias, Daniela de Laet Souza, Fabrício M. S. Oliveira, Mainá Bitar, Andrea M. Macedo, Carlos R. Machado, Marcelo V. Caliari, Glória R. Franco

**Affiliations:** ^1^Departamento de Bioquímica e Imunologia, Instituto de Ciências Biológicas, Universidade Federal de Minas GeraisBelo Horizonte, Brazil; ^2^Departamento de Patologia Geral, Instituto de Ciências Biológicas, Universidade Federal de Minas GeraisBelo Horizonte, Brazil

**Keywords:** *Schistosoma mansoni*, Y-box-binding protein 1, SMYB1, cytoplasmic antigen, vaccine candidates

## Abstract

Schistosomiasis is a neglected tropical disease, and after malaria, is the second most important tropical disease in public health. A vaccine that reduces parasitemia is desirable to achieve mass treatment with a low cost. Although potential antigens have been identified and tested in clinical trials, no effective vaccine against schistosomiasis is available. Y-box-binding proteins (YBPs) regulate gene expression and participate in a variety of cellular processes, including transcriptional and translational regulation, DNA repair, cellular proliferation, drug resistance, and stress responses. The *Schistosoma mansoni* ortholog of the human YB-1, SMYB1, is expressed in all stages of the parasite life cycle. Although SMYB1 binds to DNA or RNA oligonucleotides, immunohistochemistry assays demonstrated that it is primarily localized in the cytoplasm of parasite cells. In addition, SMYB1 interacts with a protein involved in mRNA processing, suggesting that SMYB1 functions in the turnover, transport, and/or stabilization of RNA molecules during post-transcriptional gene regulation. Here we report the potential of SMYB1 as a vaccine candidate. We demonstrate that recombinant SMYB1 stimulates the production of high levels of specific IgG1 antibodies in a mouse model. The observed levels of specific IgG1 and IgG2a antibodies indicate an actual protection against cercariae challenge. Animals immunized with rSMYB1 exhibited a 26% reduction in adult worm burden and a 28% reduction in eggs retained in the liver. Although proteins from the worm tegument are considered optimal targets for vaccine development, this study demonstrates that unexposed cytoplasmic proteins can reduce the load of intestinal worms and the number of eggs retained in the liver.

## Introduction

Schistosomiasis is the second most important neglected tropical disease causing approximately 280,000 deaths annually (King et al., 2006; Steinmann et al., 2006; Hotez et al., 2008). The disease remains endemic in several developing countries, including Brazil, where *Schistosoma mansoni* is the etiologic agent. The advent of praziquantel was essential to reduce morbidity and mortality due to schistosomiasis. However, the emergence of parasite resistant strains has been reported, raising concerns about the long-term effectiveness of this worldwide available drug (Doenhoff et al., 2002; Hotez et al., 2010). Therefore, the development of new drugs and additional control measures are essential to halt schistosomiasis dissemination. The development of a vaccine that significantly reduces parasitemia is desirable in order to allow a mass treatment with high level of protection and low costs (Chan, 1997; Katz, 1999; McManus, 1999).

Irradiated cercariae used for immunization in experimental animal models regularly induce >80% of protection (Souza et al., 1987; Lin et al., 2011; Tian et al., 2013). However, although some promising antigens have been identified and tested in clinical trials, no effective vaccine against schistosomiasis is currently available. Indeed, most of the studied anti-schistosome targets are tegumental proteins, which directly interact with the host, but consistently do not show satisfactory protection levels (McManus and Loukas, 2008). Consequently, WHO has encouraged tests with new vaccine candidates such as cytoskeletal or cytoplasmic proteins which may be used as part of a multivalent vaccine (Wilson and Coulson, 2006). Additionally, vaccines based on nuclear/cytoplasmic proteins exhibit less chance to trigger an allergic response in the vaccinated individuals (Bethony et al., 2011), as they are not directly exposed to the host immune system.

In this context, the YBPs comprise a family of proteins that are found in most living organisms (Evdokimova et al., 2006) and contain a highly conserved nucleic acid-binding domain, the cold-shock domain (CSD), which possesses great similarity to bacterial cold-shock proteins (Wistow, 1990). In addition to the CSD, proteins from this family have a variable C-terminal TAIL domain predominantly composed of basic or acid amino acids, which are responsible for either nucleic acid binding or protein-protein interactions (reviewed by Matsumoto and Bay, 2005). YBPs were originally identified as proteins that bind to DNA, RNA, and other proteins (Sommerville and Ladomery, 1996; Matsumoto and Wolffe, 1998; Valadão et al., 2002; Evdokimova et al., 2006; Dong et al., 2009; Mihailovich et al., 2010; Eliseeva et al., 2011). Subsequent studies demonstrated that YB-1, a member of this family, is a major component of ribonucleoprotein particles (mRNPs), working on pre-mRNA splicing, mRNA stability, and translation (Mihailovich et al., 2010; Brandt et al., 2012). Thus, these proteins regulate gene expression and participate in a variety of cellular processes, including transcriptional and translational regulation, induction of DNA repair, cellular proliferation, drug resistance, and stress responses to extracellular signals (Kohno et al., 2003; Mihailovich et al., 2010; Brandt et al., 2012).

In response to stress signals, including low temperatures, drugs that act on DNA, reactive oxygen species, and UV irradiation, the YB-1 protein can translocate from the cytoplasm to the nucleus and participate in gene regulation (Koike et al., 1997; Matsumoto and Wolffe, 1998; Kohno et al., 2003). One of the Y-box protein functions has been elucidated by studies of genes that are repressed in response to YB-1 overexpression in somatic cells. For example, an increase in cellular levels of the human YB-1 protein transcriptionally represses interferon-mediated activation of MHC class II genes (Ting et al., 1994). Subsequent analysis established that YB-1 stimulates the formation of single-stranded regions at the Y-box element (an inverted CCAAT motif) in a MHC class II gene promoter, preventing the loading and/or function of other transacting factors (MacDonald et al., 1995). In addition, it was reported that a synthetic protein can interact with YB-1, stimulating its translocation from the cytoplasm to the nucleus, where YB-1 binds to the promoters of collagen genes and suppresses their transcription, preventing the progression of systemic and hepatic fibrosis (Higashi et al., 2003a,b, 2011; Hasegawa et al., 2009). Currently, a number of genes involved in innate immune response processes and inflammation have been reported to be down- or up-regulated by the YB-1 protein (see the review by Raffetseder et al., 2012).

SMYB1 is a *S. mansoni* protein that belongs to the YBP family and was described by Franco et al. (1997). Due to the similarity between SMYB1 and Y-box proteins from other organisms, and the importance of these proteins in the control of gene expression, our group conducted several studies to characterize the SMYB1 protein. We reported that (i) the protein binds to double- or single-stranded DNA oligonucleotides, with a preference for sequences containing the CCAATT motif, (ii) the protein is expressed in all stages of the parasite life cycle, (iii) SMYB1 interacts with proteins involved in mRNA processing, and (iv) SMYB1 has a cytoplasmic localization (Franco et al., 1997; Valadão et al., 2002; de Oliveira et al., 2004; Rocha et al., 2013). Although the exact function of the SMYB1 protein in this parasite has not been determined, results presented by Valadão et al. (2002) and Rocha et al. (2013) suggested that, while SMYB1 may not act directly as a transcription factor, this protein may be necessary for the regulation of *S. mansoni* gene expression. These studies suggest that SMYB1 can function in the turnover, transport, and stabilization of RNA molecules, acting as RNA chaperones (Valadão et al., 2002; de Oliveira et al., 2004; Rocha et al., 2013). Although intracellular proteins are not usually the first choice of immunogens for vaccination, several extracellular *S. mansoni* proteins have been previously tested with moderate success. We have therefore decided to test SMYB1 as a vaccine candidate against this parasite. To address this matter, we have used Bioinformatics tools to investigate SMYB1 sequence composition and structural features. We have further evaluated the protective efficacy of vaccination with recombinant SMYB1 (rSMYB1) against the *S. mansoni* infection in the murine model.

## Materials and methods

### Ethics statement

Animal experiments were conducted in accordance with Brazilian Federal Law number 11,794, which regulates the scientific use of animals, and United States Institutional Animal Care and Use Committee (IACUC) guidelines. All protocols were approved by the Ethics Committee for Animal Experimentation (CETEA) at Universidade Federal de Minas Gerais under the protocol number 203/2011.

### *In silico* sequence analysis

National Center for Biotechnology Information (NCBI) BLAST (Altschul et al., 1990) searches using blastp and PSI-BLAST algorithms were performed against the UniProtKb database (The UniProt Consortium, 2013) using SMYB1 as query to identify possible SMYB1 paralogs with 90% minimal similarity. All subsequent analyses were performed for each of the three identified SMYB isoforms.

Online programs were used to assess functional characteristics of SMYB1. The InterProScan (Zdobnov and Apweiler, 2001) tool was used to recognize different protein signatures (representing protein domains, families, and functional sites) with default parameters. In addition, each SMYB protein isoform was subjected to a conserved domain search (CDS tool) (Marchler-Bauer and Bryant, 2004) from NCBI. Searches were performed against the conserved domain database (CDD v3.10; Marchler-Bauer et al., 2011) with e-values of either 0.01 or 0.001 and with or without applying the low complexity filter. The CDS analysis also points out the known DNA and RNA binding sites present within the predicted domain, by comparing to other proteins that bear the same domain.

The PredictProtein website (Rost et al., 2004) was used to generate information about the protein sequence. Several protein features can be assessed through this webserver, including amino acid composition, predicted protein binding sites and the effect of amino acid substitution. We have submitted all SMYB sequences to the PredictProtein server and retrieved specifically these three results. Protein binding sites are predicted by a machine-learning algorithm indirectly based on 3D structures to identify interacting residues using only the protein sequence as input. The effect of amino acid substitutions for each position is analyzed by exchanging the residue in each position by all other possibilities and investigating the structural/functional effect upon the protein as a whole. The impact of each point mutation is measured by a trained classifier algorithm that takes into account several features, most importantly from evolutionary information retrieved from sequence alignments. The final output of this method is presented as a heatmap, in which each column represents one position in the protein sequence and each row represents one amino acid. The neutral substitutions are colored from white to dark green, while non-neutral are colored from white to dark red. The original amino acid is marked in black.

Intrinsically disordered regions of the three SMYB isoforms were identified using Disopred (Ward et al., 2004), a trained algorithm that accurately predicts disordered regions by comparison to a dataset of protein regions that could not be solved by X-ray crystallography and, therefore, are largely flexible. False positive rate (FPR) threshold was kept in its default value of 2%.

The secretory or non-secretory nature of the protein was predicted using SignalP 4.1 (Petersen et al., 2011), which identifies signal peptides, and the SecretomeP 2.0 server (Bendtsen et al., 2004), which predicts non-classical protein secretion pathways. Both types of prediction were performed using a default setting score of 0.5. The Euk-mPLoc 2.0 (Chou and Shen, 2010) and TargetP 1.1 Servers (Emanuelsson et al., 2000) were subsequently applied to predict the subcellular locations of SMYB1. GPI-modification sites, mucin type O-glycosylation sites, and N-glycosylation sites were analyzed using the GPI Prediction Server version 3 (Eisenhaber et al., 1999), NetOGlyc 4.0 Server (Steentoft et al., 2013), and NetNGlyc 1.0 (http://www.cbs.dtu.dk/services/NetNGlyc/), respectively. Predicted serine, threonine, and tyrosine phosphorylation sites were obtained using the NetPhos 2.0 Server (Blom et al., 1999).

T and B cell epitopes were predicted based on the amino acid sequences of SMYB1, using prediction tools located at the Immune Epitope Database and Analysis Resource (IEDB-AR), which is a database of experimentally characterized immune epitopes (i.e., B and T cell epitopes) in humans, non-human primates, rodents, and other animal species (http://tools.immuneepitope.org/main/index.html). Linear B cell epitopes were predicted using programs that incorporate solvent-accessible surface area calculations and contact distances into the prediction of B cell epitope potential along the length of the protein sequence. These programs consist of the Emini Surface Accessibility Prediction (Emini et al., 1985), Kolaskar and Tongaonkar Antigenicity (Kolaskar and Tongaonkar, 1990) and the BepiPred 1.0 server (Larsen et al., 2006). To predict T cell epitopes, neural network-based prediction of proteasomal cleavage sites (NetChop) (Nielsen et al., 2005) and T cell epitopes (NetCTL and NetCTLpan) (Larsen et al., 2005; Stranzl et al., 2010) were employed.

### Cloning, expression, and purification of recombinant SMYB1

Initially, the SMYB1 cDNA (Accession no. U39883) was cloned into the pGEM-T Easy vector (Promega). The YB1fwNdeI (5′-CATATGGCGGACACTAGACC-3′) and YB1revHindIII (5′-AAGCTTGATCAGAGAATTTTAAGCGTC-3′) primers were used for SMYB1 amplification from adult worm cDNA, generating an amplification product of 675 bp. The parameters for the PCR reaction were as follows: 1 cycle at 95°C for 6 min followed by 25 cycles of 1 min at 95°C, 1 min at 58°C, 1 min at 72°C and a final cycle of 5 min at 72°C. The recombinant pGEM-SMYB1 vector was then digested with the enzymes *Nde*I and *Hin*dIII and the recovered insert was subcloned into the pET28aTEV vector, in-frame with the six histidine N-terminal (6xHis) tag. DNA sequencing was performed to confirm the presence and the correct orientation of the SMYB1 cDNA. *Escherichia coli* BL21 was transformed with the recombinant plasmid (pET28a-SMYB1) and grown in Circlegrow medium (MP Biomedicals) supplemented with kanamycin (100 μg/ml), at 37°C, 180 rpm. Bacterial growth was monitored at OD600 nm until reach 0.4–0.6 and the expression of rSMYB1 was induced by the addition of 0.5 mM IPTG. After 4 h of induction, the bacterial cells were harvested by centrifugation at 7690 g for 20 min. The pellet was resuspended in 50 mL of column buffer (20 mM sodium phosphate; 300 mM NaCl; 20 mM imidazole, pH 7.4; 10% glycerol). Lysozyme (100 μg/mL) was subsequently added, and the cells were incubated for 15 min. The cells were then subjected to 3 cycles of heat shock (−80°C/37°C), followed by three 15 s cycles of sonication (Fisher Scientific) and three rounds of centrifugation at 5940 g for 20 min. The protein was purified from the supernatant by affinity chromatography on a HisTrap HP 5 mL Ni-Sepharose column (GE Healthcare) under denaturing conditions using the ÄKTA Prime Plus Liquid Chromatography System (GE Healthcare), according to the manufacturer's instructions. Fractions containing rSMYB1 were dialyzed against Tris-NaCl buffer (50 mM Tris; 20 mM NaCl, pH 7.4), which was changed every 12 h. The dialysis was performed for 36 h at 4°C using a >12 kDa dialysis tubing cellulose membrane (Sigma Aldrich). The protein was aliquoted and stored at −80°C until use. Protein concentration was determined using Bradford's method (Bradford, 1976). The recombinant protein was used as an antigen for immunization and in immunological experiments.

### SDS-PAGE and immunoblotting

SDS-PAGE of purified rSMYB1 was performed using 12% gels, and the gels were electroblotted onto nitrocellulose membranes for 30 min at 20 V using a semi-dry system (Bio-Rad). The membranes were blocked with phosphate-buffered saline (PBS) (130 mM NaCl, 2 mM KCl, 8 mM Na2HPO4, 1 mM KH2PO4) plus 0.05% Tween 20 (PBS-T) containing 5% dry milk (p/v) for 16 h at room temperature. The membrane was subsequently incubated in 1:2000 dilutions of an anti-His antibody (GE Healthcare) and peroxidase-conjugated anti-mouse IgG (Sigma Aldrich) in PBS-T for 1 h at room temperature. After washes using PBS-T, the membrane was developed using 3,3′-diaminobenzidine (Sigma Aldrich), according to the manufacturer's protocol. After developing, the membrane was washed using distilled water and dried on filter paper.

### Immunization of mice and measurement of specific anti-rSMYB1 antibodies

Female C57BL/6 mice (*n* = 10, per group) between 6 and 8 weeks of age were obtained from the Universidade Federal de Minas Gerais (UFMG) animal facility and supplied with commercial food and water *ad libitum*. Mice were subcutaneously injected in the nape of the neck with 25 μg of rSMYB1 on days 0, 15, and 30. The vaccine was formulated with the recombinant protein emulsified in complete Freund's adjuvant (CFA) (Sigma Aldrich) for the first immunization and incomplete Freund's adjuvant (IFA) (Sigma Aldrich) for subsequent immunizations. In the control group, Tris-NaCl buffer with Freund's adjuvant was administered using the same immunization protocol.

On the tenth day after each immunization, blood was collected from each experimental group by retro-orbital bleeding. The levels of specific anti-rSMYB1 antibodies were measured by indirect ELISA. Briefly, Maxisorp 96-well microtiter plates (Nunc) were coated with 5 μg/mL rSMYB1 in carbonate-bicarbonate buffer, pH 9.6, for 16 h at 4°C. The plates were then blocked for 2 h at room temperature with 200 μl of PBS-T plus 10% fetal bovine serum (FBS) (Life Technologies) per well. The serum from each mouse was diluted 1:100 in PBS-T, and a 100-μl sample was added to each well and incubated for 1 h at room temperature. Plate-bound antibody was detected using peroxidase-conjugated anti-mouse IgG, IgG1, and IgG2a (Sigma Aldrich) diluted to concentrations of 1:5000, 1:10000, and 1:2000 in PBS-T, respectively. Color reactions were developed by the addition of 100 μL per well of 200 pmol o-phenylenediamine (OPD) (Sigma Aldrich) in citrate buffer, pH 5.0, plus 0.04% H_2_O_2_ for 10 min. The reactions were stopped with 50 μL of 5% sulfuric acid per well. The plates were read at 492 nm using an ELISA plate reader (Bio-Rad).

### Challenge infection with *S. mansoni* and worm burden recovery

Cercariae of *S. mansoni* (LE strain) were maintained routinely in *Biomphalaria glabrata* snails at the Centro de Pesquisas René Rachou - Fiocruz (CPqRR) and prepared by exposing infected snails to light for 2 h to induce shedding. Cercariae numbers and viability were determined using a light microscope prior to infection. Challenge infection was performed 10 days after the final immunization. Mice were anaesthetized with 90 mg/kg of ketamine and 10 mg/kg of xylazine. The mice abdomens were shaved and they were exposed percutaneously to 100 cercariae of *S. mansoni* in water for 1 h using the ring method (Smithers and Terry, 1965). Forty-five days after challenge (DAC), the mice were sacrificed and the adult worms were perfused from the portal veins (Fonseca et al., 2004). Two independent experiments were performed to determine protection levels and 10 mice per group were used.

Protection was calculated by comparing the number of worms recovered from each vaccinated group with its respective control group, using the following formula: PL = (WRCG − WREG) × 100/WRCG, where PL, protection level; WRCG, worms recovered from control group; and WREG, worms recovered from experimental group.

### Quantification of *S. mansoni* eggs retained in the liver

Quantification of *S. mansoni* eggs retained in the liver was performed according to the protocol described by Cheever (1968). To count the number of eggs in the liver, the organ was recovered from each experimental mouse, weighted and placed into 20 mL of a 5% KOH solution (p/v) in a 50 mL tube. Digestion occurred at room temperature for 48 h, and the samples were subsequently mixed thoroughly. The solutions were centrifuged for 3 min at 200 g, and the pellets were resuspended in 20 mL PBS and vortexed. This step was repeated three times. After the last wash, eggs were resuspended in 5 mL of 10% buffered formaldehyde in PBS and maintained at room temperature until counting. An average of three counts was obtained per 50 μL solution to estimate the number of eggs per gram of tissue. Protection was calculated by comparing the number of eggs recovered from the vaccinated group to the number of eggs recovered from its respective control group, using the same formula used for adult worms.

### Hepatic granuloma analysis

Liver sections from mice of control and vaccinated groups and infected with 100 cercariae were collected 45 days post-infection to evaluate the effect of immunization in granuloma formation. The liver sections removed from the central part of the left lateral lobe were fixed with 10% buffered formaldehyde in PBS. Histological sections were performed using microtome (4 μm) and stained in a slide with Gomory's trichromic. The granulomas were counted in Axiolab Carl Zeiss microscope using 10× objective lens. All slides were digitized by the Canon Lide 110 scanner, in 300 dpi resolution. The pixels of each histological section were fully screened, with subsequent creation of a binary image and the total area of the cut was calculated. The area of the lower cutoff was used as a minimum standard of tissue to be statistically analyzed. The results were expressed by the number of granulomas per area of liver (mm^2^). The area of granulomas was obtained through the KS300 software contained in Carl Zeiss image analyzer. Fifteen granulomas from each mouse with a single well-defined egg were randomly chosen at a microscope with 20× objective lens and scanned through a Q-Color3 microcamera (Olympus). Using a digital pad, the total area of granulomas was measured and the results were expressed in square micrometers (μm^2^).

### Humoral response against rSMYB1 and *S. mansoni* antigens after challenge

Following immunization, blood was collected using the previously described protocol (see section Immunization of Mice and Measurement of Specific anti-rSMYB1 Antibodies) at day 0 (i.e., challenge) and day 45 of infection (i.e., sacrifice). Measurements of specific anti-SMYB1, anti-*Schistosoma* worm antigen protein (SWAP), and anti-soluble egg antigen (SEA) IgG, IgG1, and IgG2a antibodies in the sera were performed using indirect ELISA, as previously described.

### Statistical analysis

Statistical analysis was performed using Student's *t*-test in the GraphPad Prism 5.0 software package (La Jolla, CA, USA).

## Results

### *In silico* analyses of SMYB1 sequence

**Figure 1:**
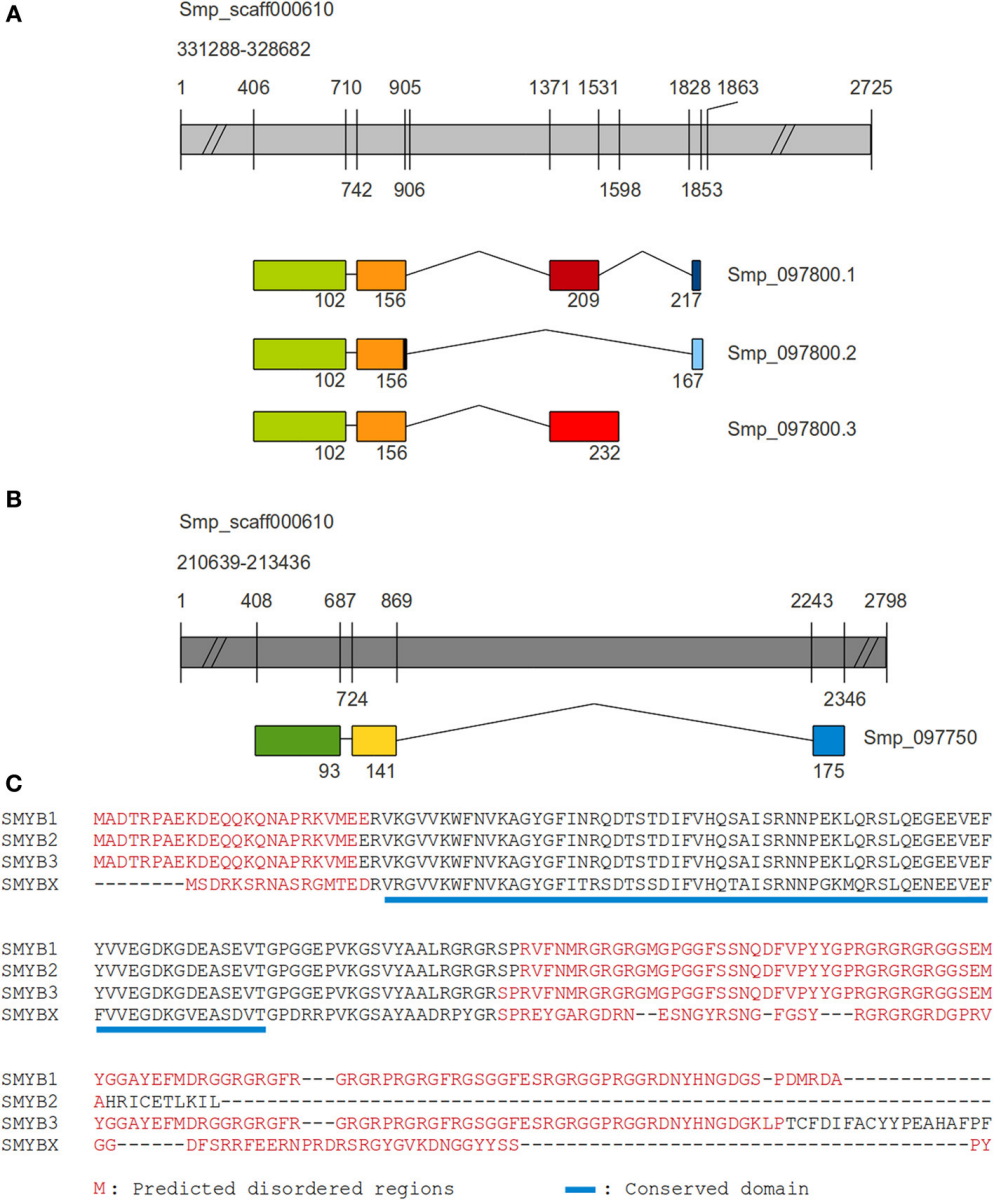
**Comparison between SMYB1 and other SMYB isoforms**. The coding sequences for all SMYB proteins were aligned to two segments of the *S. mansoni* genome scaffold 000610, in order to identify exonic and intronic portions of the genes. Genes are represented by gray bars, while exons are represented by colored boxes and introns by connecting lines. Numbers depicted on the gray bars are relative to nucleotide positions and numbers below the exons are relative to amino acid positions on the resulting protein sequence. **(A)** For SMYBX (Smp_097750), there are three exons and the third (C-terminal, blue box) one is separated from the first two (dark green and yellow boxes) by a very long intron. **(B)** Regarding its paralogs SMYB1 (Smp_097800.1) is formed by four exons and the first two (green and orange boxes) are shared with isoforms SMYB2 (Smp_097800.2, with one extra nucleotide at the end of the second exon) and SMYB3 (Smp_097800.3). While the SMYB2 isoform contains an exon (light blue box) similar to SMYB1 exon 4 (navy blue box), but longer (and in a different reading frame), SMYB3 contains a third exon (light red) which is similar to SMYB1 exon 3 (dark red), but longer. **(C)** Muscle alignment of SMYB1 (Q27277), SMYB2 (G4LXD2), SMYB3 (G4LXD0), and the SMYBX_putative (G4LXC4). Disordered regions are highlighted in red, according to Disopred predictions and the conserved domain is underlined in blue.

In *S. mansoni*, the SMYB1 protein (predicted molecular weight: 23805.20 Da, theoretical pI: 10.21) is encoded by the Smp_097800 gene, which produces three transcript isoforms: Smp_097800.1 (SMYB1), Smp_097800.2 (SMYB2), and Smp_097800.3 (SMYB3) derived from alternative splicing (Figure 1A). BLAST searches using blastp and PSI-BLAST algorithms against the UniProtKb database revealed a paralog protein in *S. mansoni* (SMYBX_putative), encoded by the Smp_097750 gene, which produces a single transcript isoform (Smp_097750.1) (Figure 1B). Global alignment shows that the SMYB proteins are much conserved (more than 90% identity). The N-terminal region (CSD) is more conserved among all sequences, consistent with the fact that all Smp_097800 derived isoforms share the first 156 amino acids, and only diverge in their C-terminal domain. Interestingly, the Smp_097750 derived isoform has an almost perfectly conserved CSD region (Figure 1C).

The InterProScan tool identified an N-terminal nucleic acid-binding OB-fold domain (IPR012340) in the SMYB isoforms (Figure 1C and Figure S1), which is found in the Y-box binding protein subfamily (PTHR11544:SF6). The presence of this domain was also confirmed by the CDS tool with high confidence (*e*-vaule of 0.001). The CDS tool has also identified a C-terminal API5 domain (apoptosis inhibitor domain 5) approximately localized between residues 140 and 200 on the longer isoforms (SMYB1 and SMYB3) although with low confidence (*e*-value of 0.01). Further analysis may confirm this as an actual conserved domain or just an artifact (Figure S1).

The prediction of intrinsically disordered regions has characterized SMYB isoforms as mostly disordered proteins. It is interesting to observe that the conserved CSD is located away from the disordered regions (Figure S1). An additional region where the disorder probability suddenly drops (flanking the residue 180) is an interesting feature to be further investigated (Figure S1). Another interesting finding regarding the disorder is its relation to protein-binding residues. For all isoforms, predicted protein binding sites range from residues 1 to ~25, ~110 to the end of the sequence and position 65, which is the only predicted binding site out of the disordered region (Figure S1 and Supplementary Material).

When observing the SNAP results presented in Figure S1, one can easily identify the first ~20 N-terminal residues as predicted to contribute very little to the structure and function of SMYB isoforms, since all simulated mutation in such positions seem to have no effect to the proteins. On the other hand, the region where the CDS domain is located is the most important and mutations in this region can easily have a negative effect to protein structure and function. This is expected, since this is the only structured region of the proteins. Accordingly, the nucleic acid binding site regions are the most conserved within this domain, since the heatmap is dark red around these sites.

SMYB1 was predicted to be located in the cytoplasm and nucleus of *S. mansoni* cells, using the Euk-mPLoc program. No cleavage sites or N-terminal presequences consistent with a mitochondrial targeting peptide or secretory pathway signal peptide were identified using the TargetP Server. In addition, the SecretomeP server revealed SecP scores below the cutoff score (0.50), indicating a low possibility of secretion by the non-classical pathway.

Additional Bioinformatics analyses of domain prediction, protein disorder, protein structure, and molecular interactions, as well as putative post-translational modifications (GPI modification, glycosylation, and phosphorylation sites) and B-cell and T-cell type epitope predictions for SMYB1 are presented and briefly discussed in the Supplementary Material (Table S1 and Figures S1, S2).

### Expression and purification of recombinant SMYB1

**Figure 2:**
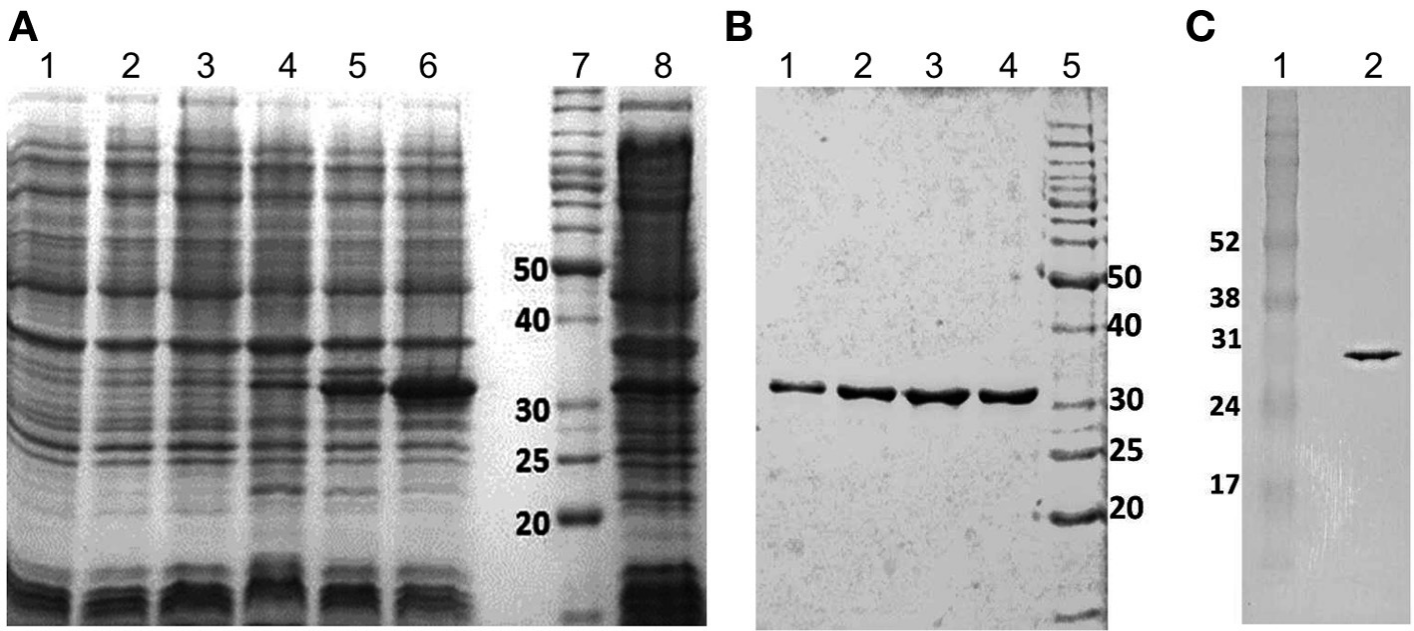
**Expression and purification of rSMYB1 as a 6xHis fusion protein. (A)** Coomasie blue-stained 12% SDS-PAGE profile of *E. coli* BL21 expressing the pET28a-SMYB1 construct. Lanes: (1) lysate of induced culture—0 h; (2) lysate of induced culture—1 h; (3) lysate of induced culture—2 h; (4) lysate of induced culture—3 h; (5) lysate of induced culture—4 h; (6) soluble fraction (SF) of induced culture lysate; (7) molecular weight marker (BenchMark Protein Ladder, Invitrogen); (8) non-soluble fraction of induced culture lysate (pellet). **(B)** Coomasie blue-stained 12% SDS-PAGE profile of the fractions of purified recombinant SMYB1. Lanes (1–4) SMYB1 fractions; lane (5) molecular weight marker (BenchMark Protein Ladder, Invitrogen). **(C)** Western blotting analysis of purified recombinant SMYB1 using an anti-His antibody (GE Healthcare). Lane (1) molecular weight marker (Amersham Full Range Rainbow Molecular Weight); lane (2) purified rSMYB1.

The SMYB1 gene was cloned into the pET28a expression vector, and the recombinant protein was successfully expressed as a 6xHis tag fusion protein. The transformed bacterial cells were treated with lysozyme, submitted to heat shock and sonication treatments, and the lysates were separated into soluble and insoluble fractions (Figure 2A). The protein was purified from the soluble fraction by affinity chromatography using His-binding columns under denaturing conditions (Figure 2B). The protein was then refolded by dialysis against Tris-NaCl buffer, with an approximate yield of 11 mg of protein/liter. The purity of the recombinant SMYB1-6xHis tag fusion protein was assessed using SDS-PAGE and Western blotting analysis with an anti-His antibody (Figure 2C), which revealed a protein of approximately 30 kDa.

### Humoral responses to rSMYB1

**Figure 3:**
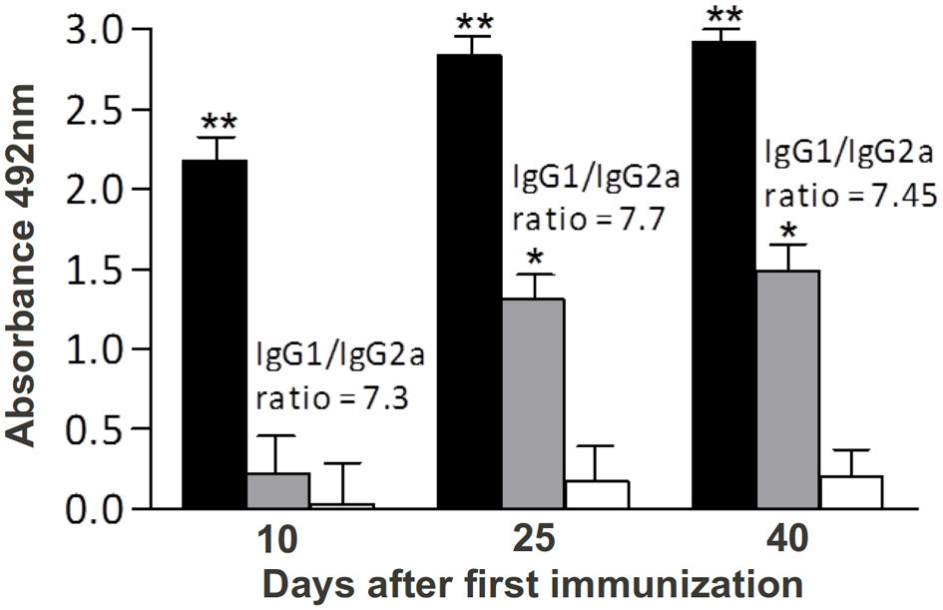
**Kinetics of specific anti-rSMYB1 IgG, IgG1, and IgG2a antibodies in sera from mice vaccinated with rSMYB1**. Sera were collected from 10 immunized mice per group prior to the first immunization and at days 10, 25, and 40 after the first immunization and assayed by ELISA. The results are presented as the mean absorbance at 492 nm for each isotype on each day of sera collection. Asterisks indicate statistically significant differences between the vaccinated and preimmune groups: ^*^p < 0.05; ^**^p < 0.01. Black bars: IgG antibody. Gray bars: IgG1 antibody. White bars: IgG2a antibody. The results are representative of two independent biological replicates.

C57Bl/6 mice were immunized with three doses of rSMYB1 formulated with Freund's adjuvant, and the level of specific anti-rSMYB1 antibodies in the sera from the immune and placebo groups was evaluated using ELISA (Figure 3). Significant levels (*p* < 0.01) of specific anti-rSMYB1 IgG antibodies were detected after the first immunization, and these antibodies remained at a high levels after the second and third immunizations. To determine the isotype of the antibody produced after immunization, IgG1 and IgG2a antibodies specific to rSMYB1 were also analyzed. The results revealed that rSMYB1 stimulates an IgG1 antibody response (*p* < 0.05) after the second dose (Figure 3). In the placebo group, no significant differences in specific IgG, IgG1, or IgG2a antibody levels were observed after immunization (data not shown).

### *S. mansoni* adult worm recovery

To determine the protective potential of rSMYB1, immunized mice were challenged with 100 *S. mansoni* cercariae. The worms were recovered by perfusion 6 weeks after challenge, and the results were expressed as the mean worm burden (mean ± *SD*) as presented in Table 1. The animals immunized with rSMYB1 in Freund's adjuvant exhibited a 26% reduction in adult worm burden recovered from the mesenteric veins when compared to the control group (*p* > 0.05). No differences in male/female proportion were observed between the placebo and immune groups (data not shown). Similar results were observed in two independent experiments.

**Table 1:** **Worm burden and protection level in mice vaccinated with the rSMYB1 protein**.

**Group**	**Worm burden (mean ± *SD*)**	**Protection**
Tris-NaCl + CFA/IFA (placebo)	51.50 ± 26.64	–
rSMYB1 + CFA/IFA (immune)	38.11 ± 10.78	26%

*CFA, complete Freund's adjuvant; IFA, incomplete Freund's adjuvant. No statistically significant differences were observed between groups (p > 0.05). The data are representative of two independent biological assays*.

### Quantification of *S. mansoni* eggs retained in the liver

**Figure 4:**
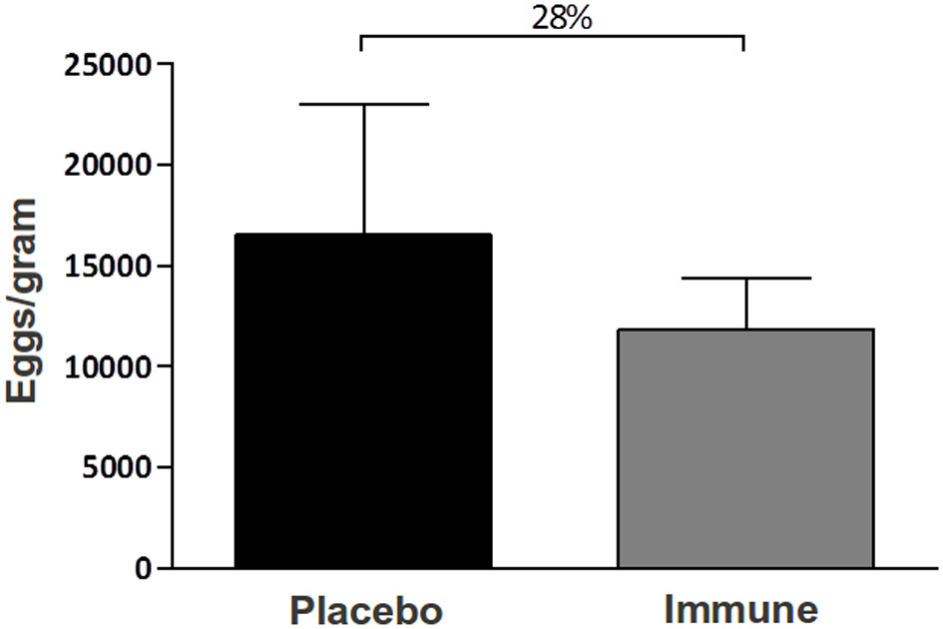
**Counting of the number of *S. mansoni* eggs retained in the liver of immunized mice**. The number of *S. mansoni* eggs in 10 mice immunized with Tris-NaCl + adjuvant (placebo group) or with rSMYB1 + adjuvant (immune group) were counted. The infection developed for 45 days prior to sacrifice. The liver was digested for 48 h in a 5% KOH solution (p/v), and the eggs were maintained in 10% buffered formaldehyde until counting. The results are presented as the mean number of eggs per gram of liver. No statistically significant differences were observed between groups (*p* > 0.05). The results are representative of two independent biological experiments.

In addition to worm counting, we evaluated the number of *S. mansoni* eggs retained in each gram of liver. The immunized group retained 28% less eggs in the liver than the placebo group (*p* > 0.05) (Figure 4). We have also measured the number of eggs layed by female adult worm recovered before and after immunization and found a 5.5% decrease in the number of eggs per female on the immunized group (the average was of 769.20 eggs/female on the placebo group against 726.69 eggs/female on the immunized group). Therefore, these result points to a combination between diminished egg production per female and decreased number of adult parasites in the host after immunization.

### Histopathological analysis

**Figure 5:**
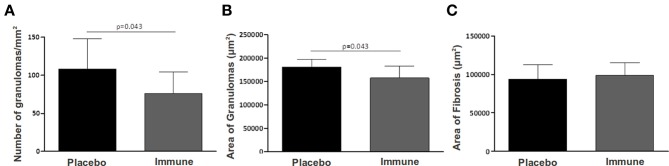
**Liver pathology measured in rSMYB1 vaccinated mice. (A)** Number of granuloma per area of liver (mm^2^), **(B)** area of granuloma (μm^2^), and **(C)** area of fibrosis (μm^2^) were measured in rSMYB1 immunized mice and control group. Data are expressed as means ± SD.

**Figure 6:**
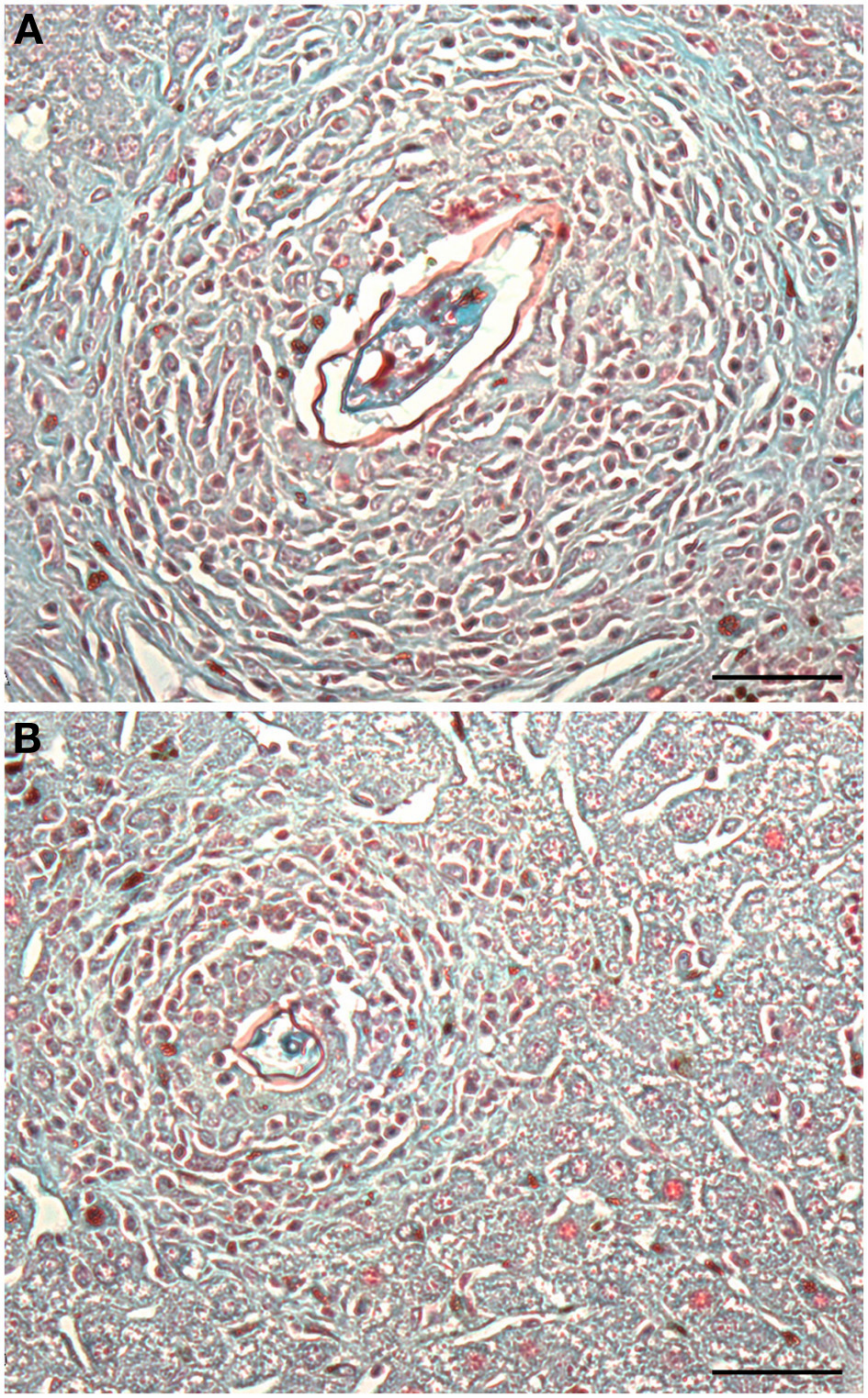
**Histological analysis of liver tissue from mice immunized with rSMYB1**. Animals were sacrificed 45 days post-infection and their livers were washed with PBS and stored in formaldehyde until histological procedures. Fixed livers were sliced using microtome (4 μm) and stained with Gomory's trichromic. **(A)** Liver from the control group and **(B)** liver from rSMYB1 immunized mice. Scale bar: 50 μm. The granulomas were counted in Axiolab Carl Zeiss microscopy using 10× objective lens.

Histopathological analysis showed significantly fewer granulomas in the liver of animals immunized with rSMYB1 (*p* < 0.05) (Figure 5A). An associated decrease in the area of granulomas in the immunized mice group compared to the placebo group (*p* < 0.05) was also observed (Figures 5B, 6). However, no significant decrease in the area of fibrosis was detected when the two groups were compared (*p* > 0.05) (Figure 5C).

### Humoral response against rSMYB1 and *S. mansoni* antigens after challenge

**Figure 7:**
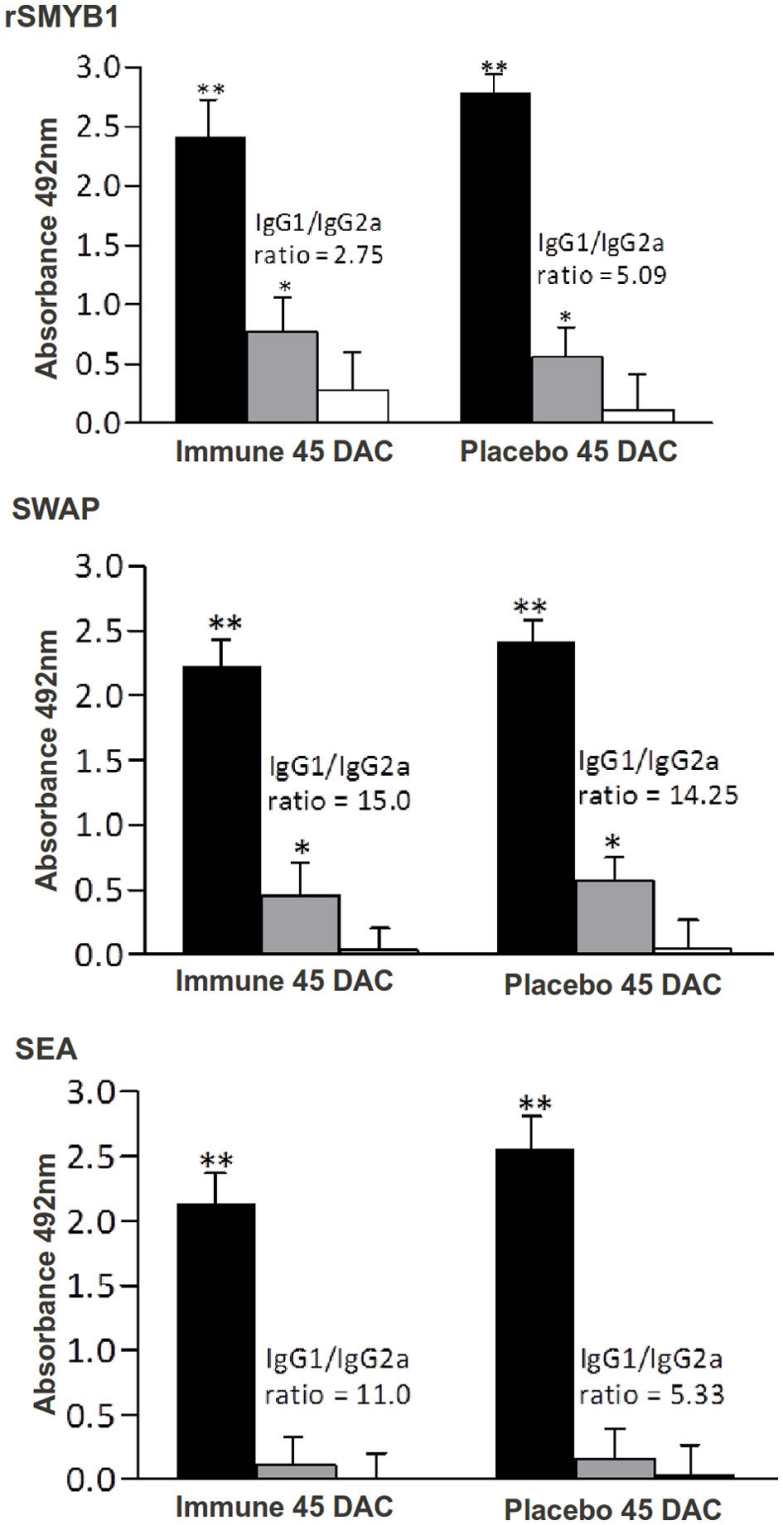
**Kinetics of specific anti-rSMYB1, anti-SWAP and anti-SEA IgG, IgG1, and IgG2a antibodies**. The presence of antibodies was measured in sera from 10 mice immunized with Tris-NaCl + adjuvant (placebo group) or with rSMYB1 + adjuvant (immune group). Sera were collected prior to sacrifice 45 days after challenge and assayed by ELISA. The results are presented as the mean absorbance at 492 nm for each immunoglobulin isotype. Asterisks indicate statistically significant differences between the vaccinated and pre-immune groups: ^*^p < 0.05, ^**^p < 0.01. DAC, days after challenge. Black bars: IgG antibody. Gray bars: IgG1 antibody. White bars: IgG2a antibody. The results are representative of two independent biological experiments.

Levels of specific antibodies produced in response to the purified rSMYB1 protein in each group of mice after challenge were determined using ELISA. Surprisingly, the levels of rSMYB1-specific IgG, IgG1, and IgG2a antibodies in the immune group decreased after the third dose of the vaccine (*p* > 0.05) (Figure 7). In contrast, the placebo group exhibited increased levels of all antibodies against the protein. No statistically significant differences were observed between the immune and placebo groups at 45 DAC (*p* > 0.05). The observed anti-rSMYB1 IgG1/IgG2a ratio decreased in the immunized mice.

To measure IgG, IgG1, and IgG2a antibodies against the specific *S. mansoni* antigens SWAP and SEA, the sera from vaccinated mice in each group were tested using ELISA (Figure 7). No specific anti-SWAP and anti-SEA IgG, IgG1, and IgG2a antibodies were detected before or during the immunizations (data not shown). After challenge, both groups developed significant levels of specific anti-SWAP and anti-SEA (Figure 7) IgG antibodies (*p* < 0.01). With respect to isotype, both groups developed a predominantly IgG1 antibody response (*p* < 0.05) against the SWAP antigen but not against the SEA antigen. No differences in IgG2a antibody response against these antigens were observed between the immune and placebo groups.

## Discussion

The long-term effective control of schistosomiasis will only occur as a result of combined vaccination and chemotherapy strategies with sanitation and public health control measures. Although evidences indicate that chemotherapy using praziquantel is effective in reducing the intensity of infection, as reinfection has been observed after chemotherapy, the use of this control strategy alone has been questioned (Wilson and Coulson, 2006). The irradiated cercarial vaccine elicits >80% protection in rodents and primates and other antigens identified in analyses of the *Schistosoma* proteome, transcriptome, glycome, and immunome, also exhibit protective potential (Oliveira et al., 2008). Nevertheless, the effectiveness of recombinant vaccines rarely exceeds 40% (McManus and Loukas, 2008; McWilliam et al., 2012), although new findings from El Ridi and collaborators (El Ridi and Tallima, 2013; El Ridi et al., 2014) are promising and depict a decrease of ~70–80% in worm burden using papain as adjuvant and focusing on *S. mansoni* cysteine peptidases as antigens. To date, vaccine candidates have been assessed using omics-derived high throughput approaches, such as proteomics, immunomics, and vaccinomics with promising results (DeMarco and Verjovski-Almeida, 2009; Loukas et al., 2011; McWilliam et al., 2012).

Many studies focus on tegument proteins as potential drug/vaccine targets because the tegument is a dynamic layer that represents the primary host-parasite interface and has close proximity to the host blood and immune system (Jones et al., 2004; Pearce and Freitas, 2008; DeMarco and Verjovski-Almeida, 2009; Han et al., 2009; Loukas et al., 2011). Other studies focus on excretory/secretory (ES) proteins, molecules known to be released from live worms in the tissue culture and that may be secreted into host tissues as the parasites move along the host body, feed, and produce eggs (Loukas et al., 2011). According to McManus and Loukas (2008), the apical membrane proteins expressed on the surfaces of the schistosomulum and the adult worm are the preferred vaccine targets. Therefore, the use of extracellular antigens for vaccine production is accompanied by inherent problems, for instance, the difficulty to produce recombinant proteins, since the majority of these antigens is processed through the classical secretory pathway and is subject of complex post-translational modifications, (e.g., glycosylation, specific processing, and disulfide bonds formation). Additionally, most antigens tested in WHO trials and by other groups are cytosolic or cytoskeletal components (e.g., paramyosin, Sm14, and GST) (Wilson and Coulson, 2006; McManus and Loukas, 2008; Oliveira et al., 2008). To our knowledge no study exploring the potential of a nucleic acid-binding protein as a *S. mansoni* vaccine candidate has been published. This is the first attempt to characterize such a protein as an antigen and to evaluate its protective efficacy as a vaccine against *S. mansoni* infection in the murine model.

In 2005, Carl and collaborators have stated that most nuclear systemic autoantigens contain long regions of structural disorder. They have studied properties of intrinsically disordered proteins in order to make connections linking disorder to antigenicity. The authors state that the amino acid composition of disordered regions (usually rich in Arg, Gly, Ser, Pro, Glu, Lys, Gln, and Ala residues) leads to a highly charged and low complexity molecule, typical properties of autoantigens (Plotz, 2003). Another property of autoantigens is their capacity to bind nucleic acids, as described by Plotz and cited by Carl and collaborators. Additionally, Plotz listed phosphorylation as a strong feature of autoantigens. All of these factors, namely enrichment in six of the listed amino acids (Arg, Gly, Ser, Pro, Glu, Lys), low complexity regions (such as repetitive sequence patterns), nucleic acid binding capacity and the abundance of phosphorylation sites (10 predicted) can be observed in the SMYB1 protein, thus corroborating its putative antigenic potential.

Although the Euk-mPLoc program predicted the SMYB1 localization in the cytoplasm and nucleus of cells, our group has previously demonstrated that this protein is predominantly located in the cytoplasm of cells from different life cycle stages of *S. mansoni*, suggesting that SMYB1 is probably acting in RNA metabolism in the cytoplasm. We also showed the presence of SMYB1 near the tegument in adult worms proposing an action on the translational regulation of tegument proteins (Rocha et al., 2013). Intrinsically disordered proteins have recently been characterized as the prevalent type of RNA and protein chaperones (Tompa and Csermely, 2004). Accordingly, it has been shown that YBPs and other cold-shock proteins typically act as chaperones that maintain mRNA in a single-stranded conformation to sustain the expression of genes that are necessary for cell growth, proliferation, and transformation (Jiang et al., 1997; Matsumoto and Wolffe, 1998; Salvetti et al., 1998; Tanaka et al., 2004; Evdokimova et al., 2006). YBPs are thought to play roles in a wide variety of responses to environmental stresses (Kohno et al., 2003). As such, SMYB1 localization in the cytoplasm of tegumental cells reinforces its importance as a protein that acts responding to the stressing host environment.

Molecules that contain signal peptides or signal anchors are predicted to be excreted, secreted or membrane-anchored, directly interacting with the host immune system and, as stated above, constitute relevant targets for schistosome vaccines. The combined Bioinformatics results obtained in this study suggest that the SMYB1 protein is not secreted. However, Frye et al. (2009) reported that human YB-1 is secreted from cells during inflammatory stress after treatment with lipopolysaccharide, hydrogen peroxide or TGFβ. In these cases, YB-1 is secreted not via the classical mechanism of protein secretion (i.e., via the Golgi apparatus and endoplasmic reticulum) but by a non-classical mechanism inside endolysosomal vesicles (Frye et al., 2009; Eliseeva et al., 2011). The question of whether SMYB1 is secreted or not needs further experimental investigation.

We reported here the successful cloning of SMYB1 cDNA into the pET28a vector and the expression of rSMYB1 in the soluble fraction of bacterial lysates. The discrepancy between the ~30 kDa protein molecular mass value calculated from SDS-PAGE and the ~24 kDa protein molecular mass value predicted from the cDNA is typical of Y-box proteins and related to the anomalous electrophoretic properties of these proteins (Deschamps et al., 1992) or to post-translational modification, such as phosphorylation (Salvetti et al., 1998). We subsequently evaluated the antigenicity of the protein by investigating the murine humoral immune response to rSMYB1 and the impact of its immunization on adult worm and egg burden in mice challenged with 100 cercariae of *S. mansoni*. Recent data suggested that the establishment of a robust humoral response is likely the key for generating maximal immunity against schistosomes (Wynn and Hoffmann, 2000). A primary obstacle to the development of a schistosome vaccine is the lack of available knowledge concerning the type of immune response that should be induced. In the irradiated cercariae vaccination model, above 80% protection can be granted by a Th1, a Th2, or a mixed Th1/Th2 immune response (Wynn and Hoffmann, 2000). However, with respect to recombinant proteins, Th1-inducing antigens have been reported to confer protection against *Schistosoma* infection in the mouse model (Jankovic et al., 1996; Mountford et al., 1996; Zhang et al., 2001; Fonseca et al., 2004; Varaldo et al., 2004; Li et al., 2005; Cardoso et al., 2008; Garcia et al., 2008).

In this study, C57Bl/6 mice immunized with rSMYB1 exhibited high levels of specific anti-SMYB1 IgG antibodies that emerged after the first immunization. Specific anti-SMYB1 IgG1 antibodies predominated over IgG2a antibodies, particularly after the second immunization. However, the IgG1/IgG2a ratio was reduced after the last immunization (i.e., during the challenge period). Antibody levels correlated with protective efficacy in our study. The antibody levels developed by mice immunized with rSMYB1 reduced in 26% the number of adult worm burden and in 28% the eggs/granuloma trapped in the liver. A critical issue in vaccine design is the use of an appropriate adjuvant to induce the suitable immune response. Although the CFA adjuvant, which triggers a Th1 response, cannot be used in humans (Heegaard et al., 2011), it is widely utilized in initial immunization trials. Further experiments combining rSMYB1 with suitable adjuvant formulations for use in humans should be performed. In this sense, an interesting strategy would be to use papain as adjuvant, given that recently published articles have described very high protection rates related to the use of such molecule in vaccine candidates (El Ridi and Tallima, 2013; El Ridi et al., 2014).

*S. mansoni* adult worms live in the blood essentially unrecognized for many years, whereas schistosome eggs are a prominent target of the host immune response. In the first weeks of murine *S. mansoni* infection, a Th1 immune response is observed and the eggs deposited in the blood vessels by females that pass to the endothelial barrier and become trapped in the liver are immediately targeted by recruited immune cells that consist primarily of T-cells, eosinophils, and macrophages (Pearce and MacDonald, 2002; Wynn et al., 2004). Histopathology results show that in the initial phase of infection vaccination with SMYB1 seems to interfere with cell recruitment and migration in the liver. Consequently, the resulting granulomas, although presenting the same area of fibrosis, were fewer when compared to unvaccinated animals, showing the protective potential of the protein in the initial liver pathology.

Although tegument proteins are considered the main targets for vaccine development (Bergquist et al., 2002; McManus and Loukas, 2008), this study demonstrates that a cytoplasmic protein has the potential to be used as an immunogen, as we showed that SMYB1 could reduce the load of intestinal worms and eggs retained in the liver when it was used in vaccination trials and also that the protection levels achieved by SMYB1 are comparable to those obtained with other tegument and cytoskeleton proteins.

## Author's contributions

Sílvia R. C. Dias, Mariana Boroni, Thomaz L. Dias, Daniela de Laet Souza, Fabrício M. S. Oliveira, Elizângela A. Rocha, Mainá Bitar, Andrea M. Macedo, Carlos R. Machado, Marcelo V. Caliari, and Glória R. Franco contributed to the conception and design of the experiments; Sílvia R. C. Dias, Mariana Boroni, Thomaz L. Dias, Daniela de Laet Souza, Fabrício M. S. Oliveira, Elizângela A. Rocha, Mainá Bitar, Andrea M. Macedo, Carlos R. Machado, Marcelo V. Caliari, and Glória R. Franco performed data acquisition, analysis, and interpretation of the results; Sílvia R. C. Dias, Mariana Boroni, Mainá Bitar, Fabrício M. S. Oliveira, Marcelo V. Caliari, and Glória R. Franco contributed to drafting the manuscript; Sílvia R. C. Dias, Mariana Boroni, Mainá Bitar, Fabrício M. S. Oliveira, Andrea M. Macedo, Carlos R. Machado, Marcelo V. Caliari, and Glória R. Franco critically revised intellectual content of the work.

### Conflict of interest statement

The authors declare that the research was conducted in the absence of any commercial or financial relationships that could be construed as a potential conflict of interest.
