# Supplementary material for: Evaluation of the Schistosoma mansoni Y-box-binding protein (SMYB1) potential as a vaccine candidate against schistosomiasis
Source: Front Genet. 2014 Jun 11;5:174. doi: 10.3389/fgene.2014.00174 (PMC4052899; doi:10.3389/fgene.2014.00174)
Supplement: Supplementary file 1 [file DataSheet1.PDF]

Bioinformatic Analysis of SMYB1

Conserved Domains Annotation

We have searched for conserved domains across the protein sequences using the CDS tool. Regardless of the e-value threshold used, the N-terminal CSD was shown to be a conserved domain and approximately placed between residues 25 and 95 in all three isoforms. On the other hand, the presence of a C-terminal API5 domain (apoptosis inhibitor domain 5) approximately localized between residues 140 and 200 was suggested for the longer isoforms (SMYB1 and SMYB3) when the e-value cutoff was of 0.01. When we lowered this cutoff to 0.001, or applied a low complexity filter, the results did not indicate the presence of the API5 domain in any protein isoform and for this reason we are not sure if this is an actual conserved domain or just an artifact **(Figure S1)**.

Protein Structure and Disorder Assessment

The prediction of intrinsically disordered regions has characterized SMYB isoforms as mostly disordered proteins. It is interesting to observe that the conserved CSD is located away from the disordered regions **(Figure S1)**. Part of the putative API5 domain is located in a region where the disorder probability is lower than flanking regions and might indicate this domain exists in this location, since structured domains are not observed in highly disordered regions. Even if the API5 domain is proven to be absent in SMYB proteins, the region where the disorder probability suddenly drops (flanking the residue 180) is an interesting feature to be further investigated **(Figure S1)**. Another interesting finding regarding the disorder is its relation to protein-binding residues. This may be due to surface accessibility, combined with other amino acid attributes. Results are generally the same for all three protein isoforms. Predicted protein binding sites range from residues 1 to ~25 and ~110 to the end of the sequence. There is only one binding site predicted away from disordered regions, approximately at amino acid 63 (**Figure S1**).

Contribution of Individual Amino Acids for Protein Structure and Function

When observing the heatmap in **Figure S1**, one can easily identify the first ~20 N-terminal residues as contributing very little to the structure and function of SMYB isoforms, since any simulated mutation in such positions are predicted to have no effect to the proteins. On the other hand, the region where the CDS domain is located is the most important and changes in amino acids in this region can easily have a negative effect to protein structure and function. This is expected, since this is the only structured region of the proteins. Interestingly, the nucleic acid binding site regions are the most conserved within this domain, since the heatmap is dark red around these sites. On the other hand, the region where the solely protein binding site of the cold-shock domain is placed (residue 63) seems to be less conserved and tolerate mutations.

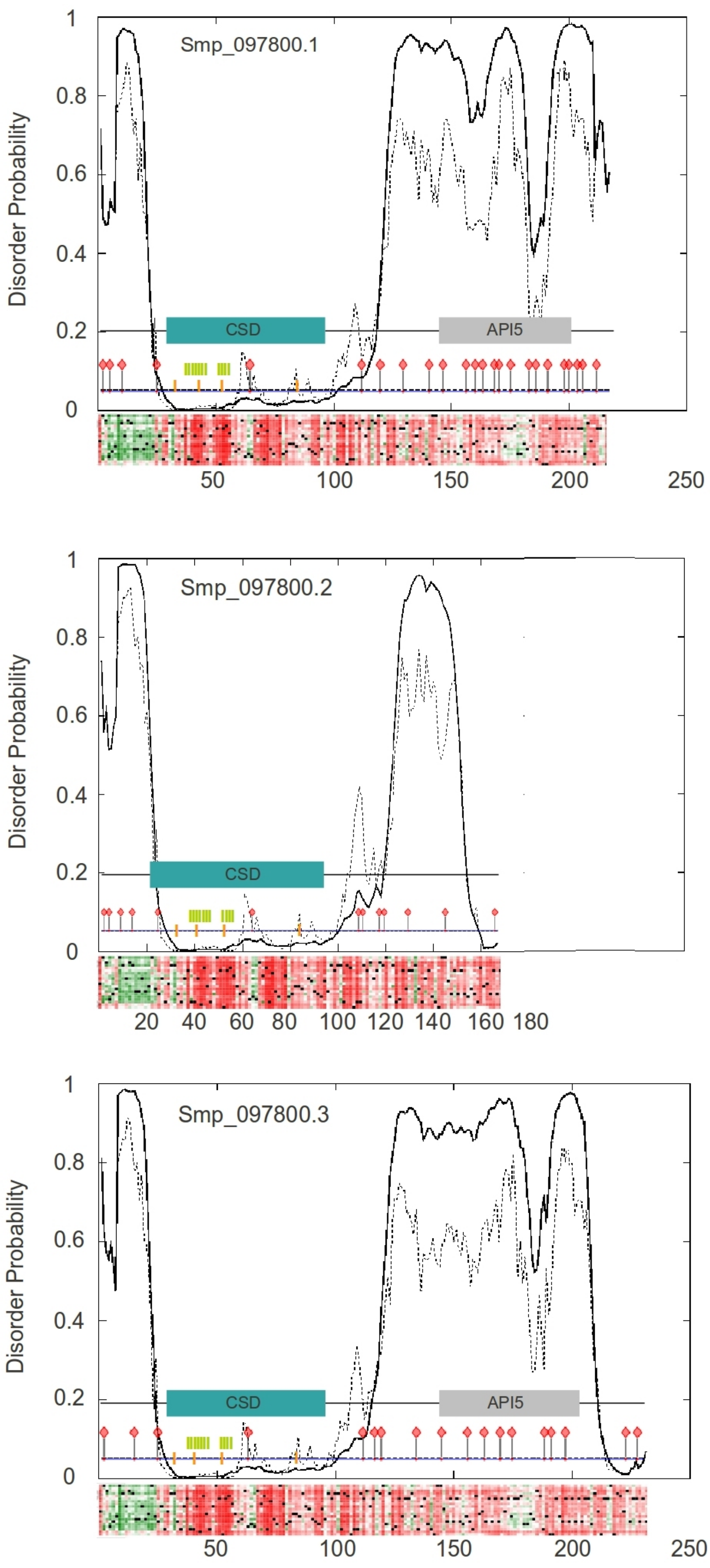

Figure S1. Sequence features of SMYB isoforms. This figure depicts Disopred, CDS and PredictProtein results for the three SMYB isoforms. Each graph shows the probability of disorder (y-axis) per residue (x-axis) for a different isoform, where the finely dashed trace is the raw output and the solid black line is the filtered output. The FPR threshold was set in 2% (the 5% cutoff is shown as a dashed blue line). Residues predicted as disordered are the ones with filtered values greater than cutoff. Within each graph the predicted domain architecture and binding sites are represented. Each predicted domain is shown as a box (turquoise for the CSD and grey for the putative API5 domain). Disordered regions are represented as lines. SMYB2 lacks the putative API5 domain. Protein-protein binding sites predicted by PredictProtein are represented as red diamonds across the protein sequence, while DNA and RNA binding sites annotated according to CDS results are shown as orange and green bars, respectively. Below each graph a heatmap generated by the PredictProtein using the SNAP algorithm is plotted. Each column corresponds to one position in the protein sequence, the black square represents the amino acid that occupies each position in SMYB proteins and each line represents the substitution of such residue by a different one. If the amino acid change is predicted to have a neutral effect, it is colored from white to dark green and if it has a non-neutral effect it is colored from white to dark red.

Post-Translational Modifications:

Concerning putative post-translational modifications, no potential GPI-modification or N-glycosylation sites were found in SMYB1 using the GPI Prediction Server and NetNGlyc 1.0. However, the NetOGlyc Server predicted five mucin-type GalNAc O-glycosylation site, at the residues Thr4, Ser103, Ser114, Ser187, Ser192. In addition, the results obtained using the NetPhos Server revealed ten possible phosphorylation sites: six at serine residues at positions 59, 69, 114, 154, 187 and 211, one at a threonine residue at position 47, and three at tyrosine residues at positions 79, 161 and 205.

Epitope Prediction:

The SMYB1 protein was predicted to have a large number of epitopes. By combining the three methods for predicting linear B-cell type epitopes, we predicted 12 distinct peptide sequences that may act as epitopes (**Table S1**). Three identified peptides (DTRPAEKDEQQKQNAP, LRNNPEK and ASEVTGP) were recognized using at least two methods. The T-cell epitope prediction using the NetChop server and the NetCTL-1.2 server, identified 59 proteasome cleavage sites and 3 class I MHC ligands (GSEMYGGAY, SSNQDFVPY and STDIFVHQS), respectively, in the protein (**Figure S2A and S2B**).

Table S1. Predicted linear B-cell epitopes.

Method	N°	Start Position	End Position	Peptide	Peptide Length
Emini Surface Accessibility Prediction	1	3	18	DTRPAEKDEQQKQNAP	16
	2	60	66	LRNNPEK	7
	3	200	206	GGRDNYH	7
Kolaskar & Tongaonkar Antigenicity	1	26	42	VKGVVKWFNVKAGYGFI	17
	2	49	58	TDIFVHQSAI	10
	3	76	83	VEFYVVEG	8
	4	89	95	ASEVTGP	7
	5	97	109	GEPVKGSVYAALR	13
	6	135	144	NQDFVPYYGP	10
Bepipred Linear Epitope Prediction	1	1	21	MADTRPAEKDEQQKQNAPRKV	21
	2	44	48	RQDTS	5
	3	58	75	ISRNNPEKLQRSLQEGEE	18
	4	83	103	GDKGDEASEVTGPGGEPVKGS	21
	5	110	115	GRGRSP	6
	6	123	158	RGRGMGPGGFSSNQDFVPYYGPRGRGRGRGGSEMYG	36

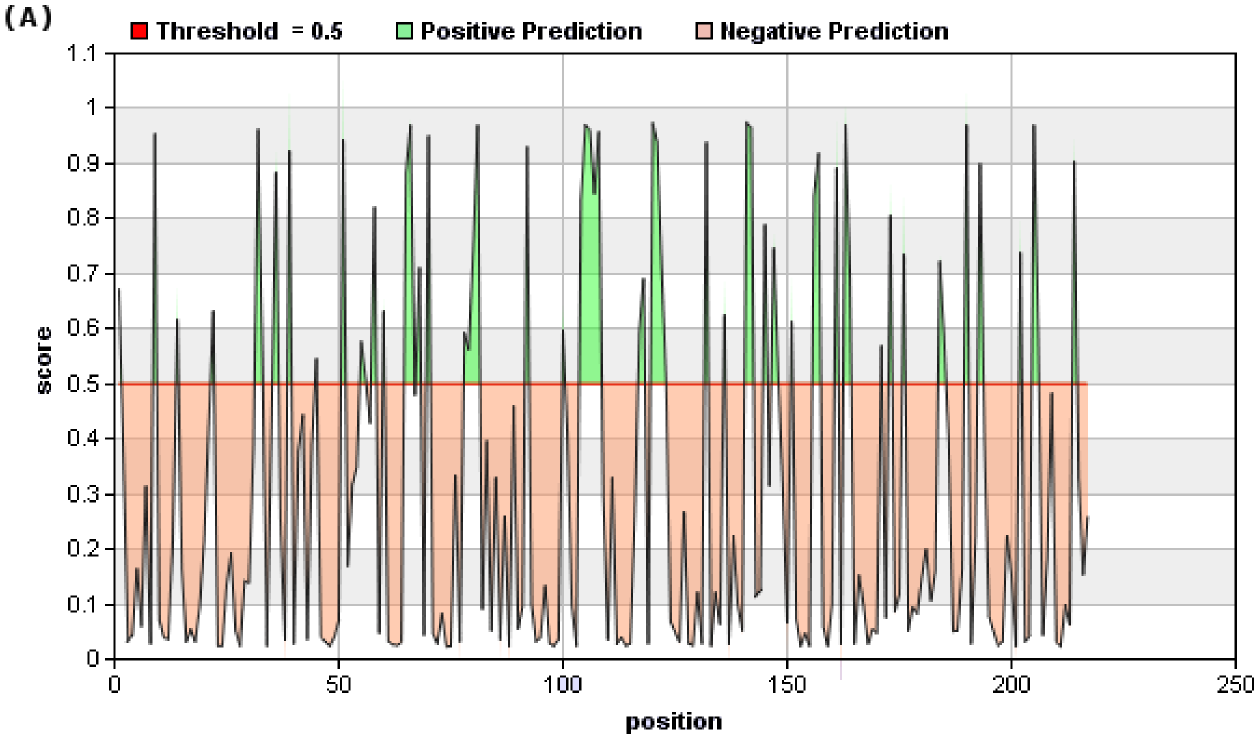

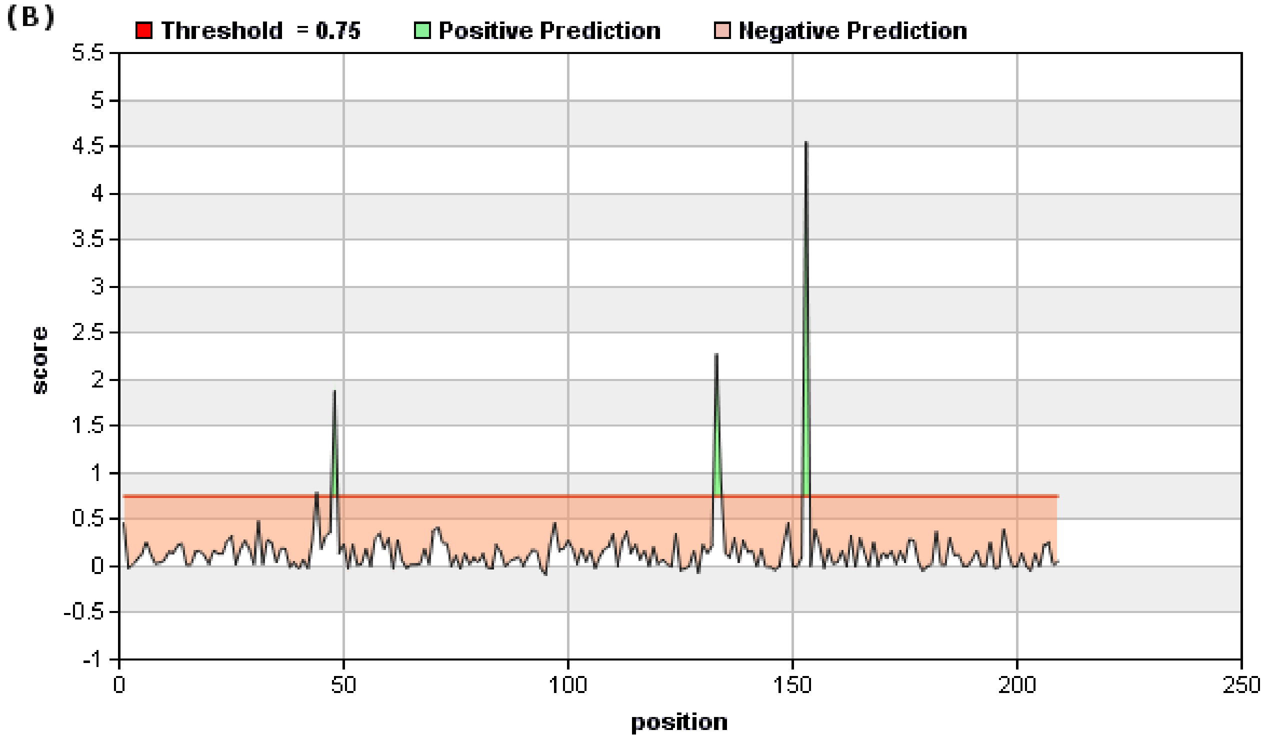

Figure S2. Predicted T-cell epitopes for SMYB1. (A) Predicted continuous epitopes, presented as a chart of NetChop score vs. residue position. **(B)** Predicted continuous epitopes, presented as a chart of NetCTL score vs. residue position. Positive predictions are displayed in green, and predictions below the threshold value (0.5) are displayed in red.
